# Supplementary material for: The Evolution and Characterization of the RNA Interference Pathways in Lophotrochozoa
Source: Genome Biol Evol. 2024 May 7;16(5):evae098. doi: 10.1093/gbe/evae098 (PMC11114477; doi:10.1093/gbe/evae098)
Supplement: evae098_Supplementary_Data [file evae098_supplementary_data.zip › Supplementary_materials.zip!/selected_miRNAs/NoGe_selected_miRNAs/mirdeep_pdf_files/NMRB01000824.1_Notospermus_geniculatus_strain_Ushimado_scaffold824,_whole_genome_shotgun_sequence_21799.pdf]

Provisional ID	:	NMRB01000824.1_Notospermus_geniculatus_strain_Ushimado_scaffold824,_whole_genome_shotgun_sequence_21799
Score total	:	121087.7
Score for star read(s)	:	3.9
Score for read counts	:	121083.1
Score for mfe	:	1.3
Score for randfold	:	0
Score for cons. seed	:	-0.6
Total read count	:	237511
Mature read count	:	237446
Loop read count	:	0
Star read count	:	65

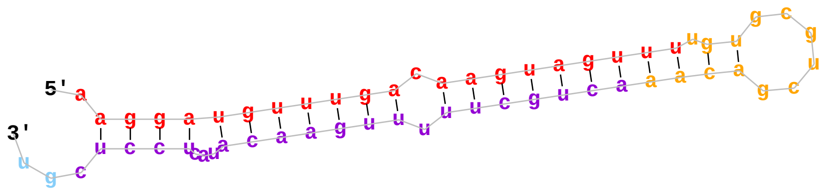

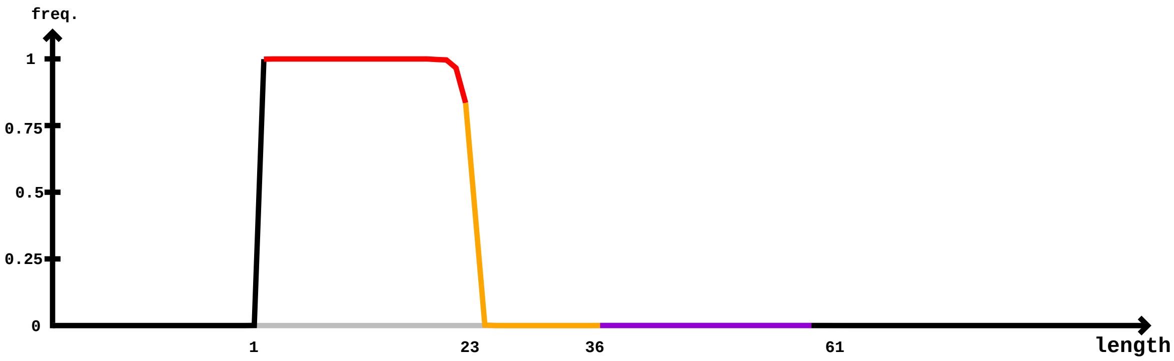

Mature **Star**

5'- aaccuugguauaugaaacguaaggauguuugacaaguaguuuugugcgucgacaaacugcuuuuugaacauacuccucguuucuuuaccgagucugcuuauugcuucacgug -3'	obs		
aaccuugguauaugaaacguaaggauguuugacaaguaguuuugugcgucgacaaacugcuuuuugaacauacuccucguuucuuuaccgagucugcuuauugcuucacgug	exp		
...(((((((...((((((..(((((((((((.(((((((((.((......))))))))))).)))))))...))))))))))..)))))))...((.....))........	reads	mm	sample
...................uaaggauguuugacaaguaguu...	2	0	Mm2
...................Caaggauguuugacaaguaguuu..	17	1	Mm2
...................uaaggauguuugacaaguaguuu..	1	0	Mm2
....................aaggauguuugacaagua..	22	0	Mm2
....................aaggauguuugacaaguU..	1	1	Mm2
....................aaggauguuugacaaguag...	13	0	Mm2
....................aaggauguuugacaaguUg...	1	1	Mm2
....................aaggCuguuugacaaguagu..	1	1	Mm2
....................aaggauguuugacaaguCgu..	1	1	Mm2
....................aGggauguuugacaaguagu..	2	1	Mm2
....................aaggauguuugacaaguUgu..	1	1	Mm2
....................Gaggauguuugacaaguagu..	1	1	Mm2
....................aaggauguuugacaaguGgu..	2	1	Mm2
....................aaggauguuugacaaguagu..	445	0	Mm2
....................Uaggauguuugacaaguagu..	2	1	Mm2
....................aaggauguuugacaagAagu..	1	1	Mm2
....................aaggauguuugacaGguaguu...	1	1	Mm2
....................aCggauguuugacaaguaguu...	1	1	Mm2
....................aaggauguuuCacaaguaguu...	3	1	Mm2
....................aaggauguuugacaaguUguu...	9	1	Mm2
....................aaggauguuugacaaguGguu...	7	1	Mm2
....................aaUgauguuugacaaguaguu...	2	1	Mm2
....................aUggauguuugacaaguaguu...	2	1	Mm2
....................aaggauguuugacaagCaguu...	16	1	Mm2
....................aNggauguuugacaaguaguu...	1	1	Mm2
....................aaggauguCugacaaguaguu...	1	1	Mm2
....................aaggauguuugUcaaguaguu...	1	1	Mm2
....................aaggauguuuAacaaguaguu...	7	1	Mm2
....................Gaggauguuugacaaguaguu...	9	1	Mm2
....................aaggauguuugacaaguaguC...	1	1	Mm2
....................aaggauguuugacaaguaguG...	4	1	Mm2
....................aaggauguuugGcaaguaguu...	2	1	Mm2
....................Naggauguuugacaaguaguu...	1	1	Mm2

Mature Star

```
aaccuugguauaugaaacguaaggauguuugacaaguaguuuugugcgucgacaaacugcuuuuugaacauacucucguucuuuaccgagucugcuuauugcuucacgug
....................aaggauguuuUacaaguaguu.....................................................................	3	1	Mm2
....................aaggauguuugacUaguaguu.....................................................................	2	1	Mm2
....................aaggauguuugacaagAaguu.....................................................................	8	1	Mm2
....................aaggauguuugacGaguaguu.....................................................................	1	1	Mm2
....................aaggGuguuugacaaguaguu.....................................................................	1	1	Mm2
....................aaggauguuugacaUguaguu.....................................................................	1	1	Mm2
....................aaggauguuugacaaguCguu.....................................................................	8	1	Mm2
....................aaggauguuugacaagGaguu.....................................................................	9	1	Mm2
....................Caggauguuugacaaguaguu.....................................................................	2	1	Mm2
....................aaggCuguuugacaaguaguu.....................................................................	1	1	Mm2
....................Uaggauguuugacaaguaguu.....................................................................	11	1	Mm2
....................aaggauguuugacaaguaguu.....................................................................	3713	0	Mm2
....................aaggaCguuugacaaguaguu.....................................................................	1	1	Mm2
....................aaggauguuugacaaguaguA.....................................................................	6	1	Mm2
....................aGggauguuugacaaguaguu.....................................................................	18	1	Mm2
....................aaggauguuugaUaaguaguuu....................................................................	4	1	Mm2
....................aagAauguuugacaaguaguuu....................................................................	3	1	Mm2
....................aaggauguuugacaagGaguuu....................................................................	95	1	Mm2
....................aaggauguuGgacaaguaguuu....................................................................	1	1	Mm2
....................aaggauguuugacaagCaguuu....................................................................	120	1	Mm2
....................aaggGuguuugacaaguaguuu....................................................................	5	1	Mm2
....................aaggauguuugacCaguaguuu....................................................................	1	1	Mm2
....................aaggauCuuugacaaguaguuu....................................................................	1	1	Mm2
....................aaggauguAugacaaguaguuu....................................................................	2	1	Mm2
....................aaggauguuugacaaguaguGu....................................................................	4	1	Mm2
....................aaggauguuugacaaguaguCu....................................................................	4	1	Mm2
....................aagCauguuugacaaguaguuu....................................................................	2	1	Mm2
....................Naggauguuugacaaguaguuu....................................................................	15	1	Mm2
....................aaggauguuugacaaguagGuu....................................................................	3	1	Mm2
....................aaUgauguuugacaaguaguuu....................................................................	5	1	Mm2
....................aagUauguuugacaaguaguuu....................................................................	2	1	Mm2
....................aaggauguuugacaaguCguuu....................................................................	110	1	Mm2
....................aaggauguuugCcaaguaguuu....................................................................	1	1	Mm2
....................aaggCuguuugacaaguaguuu....................................................................	3	1	Mm2
....................aaggauguGugacaaguaguuu....................................................................	2	1	Mm2
....................aaggauguuugGcaaguaguuu....................................................................	5	1	Mm2
....................aUggauguuugacaaguaguuu....................................................................	4	1	Mm2
....................aaggauguuugacaagAaguuu....................................................................	70	1	Mm2
....................aaggaugGuugacaaguaguuu....................................................................	2	1	Mm2
....................aaggauguuugacaaguagCuu....................................................................	7	1	Mm2
....................aaggauguuugacaUguaguuu....................................................................	8	1	Mm2
....................aaAgauguuugacaaguaguuu....................................................................	3	1	Mm2
....................aGggauguuugacaaguaguuu....................................................................	182	1	Mm2
....................aaggauguuugacaaguaguuC....................................................................	2	1	Mm2
....................aaggauguuugacaaguGguuu....................................................................	91	1	Mm2
....................aaggauguuugacaaguaguAu....................................................................	1	1	Mm2
....................aaggauguuuUacaaguaguuu....................................................................	28	1	Mm2
....................aaggaAguuugacaaguaguuu....................................................................	1	1	Mm2
....................aaggauguuugacaaguUguuu....................................................................	74	1	Mm2
....................aaggauguuugacaaguagAuu....................................................................	4	1	Mm2
....................aaggauguCugacaaguaguuu....................................................................	1	1	Mm2
....................aaggaNguuugacaaguaguuu....................................................................	4	1	Mm2
....................Uaggauguuugacaaguaguuu....................................................................	51	1	Mm2
....................aaggauguuugacaaguaguuA....................................................................	39	1	Mm2
....................aNggauguuugacaaguaguuu....................................................................	1	1	Mm2
....................aaggauNuuugacaaguaguuu....................................................................	2	1	Mm2
....................aaggauguuugacaaguaguuu....................................................................	32256	0	Mm2
....................aaggauguuugacaaUuaguuu....................................................................	4	1	Mm2
....................aaggauguuCgacaaguaguuu....................................................................	4	1	Mm2
....................aaggaugCuugacaaguaguuu....................................................................	2	1	Mm2
....................Gaggauguuugacaaguaguuu....................................................................	69	1	Mm2
....................aCggauguuugacaaguaguuu....................................................................	3	1	Mm2
....................aaggauguuAgacaaguaguuu....................................................................	4	1	Mm2
....................aaggauAuuugacaaguaguuu....................................................................	3	1	Mm2
....................aaggauguuugacaaguaguuG....................................................................	25	1	Mm2
....................aaggauguuugacGaguaguuu....................................................................	6	1	Mm2
....................aaggaugAuugacaaguaguuu....................................................................	5	1	Mm2
....................aaggauguuugacUaguaguuu....................................................................	1	1	Mm2
....................aaggauguuugacaaguaUuuu....................................................................	2	1	Mm2
....................aaggauUuuugacaaguaguuu....................................................................	1	1	Mm2
```

Mature Star

```
aaccuugguauaugaaacguaaggauguuugacaaguaguuuugugcgucgacaaacugcuuuuugaacauacuccucguuucuuuaccgagucugcuuauugcuucacgug
....................aaggUuguuugacaaguaguuu......................................................................	5	1	Mm2
....................aaggaCguuugacaaguaguuu......................................................................	2	1	Mm2
....................aaggauguuugacaaAuaguuu......................................................................	2	1	Mm2
....................aaggauguuuAacaaguaguuu......................................................................	60	1	Mm2
....................aaggauguuuCacaaguaguuu......................................................................	30	1	Mm2
....................aaggauguuugUcaaguaguuu......................................................................	15	1	Mm2
....................aaggauguuugacaGguaguuu......................................................................	4	1	Mm2
....................Caggauguuugacaaguaguuu......................................................................	3	1	Mm2
....................aaggauguuugacaaguaguuuC.....................................................................	1	1	Mm2
....................aaggauguuuAacaaguaguuuu.....................................................................	4	1	Mm2
....................aaggauguuugacaagAaguuuu.....................................................................	3	1	Mm2
....................aaggauguuugacaaguaguuuu.....................................................................	1678	0	Mm2
....................aaggauguuugacaaguUguuuu.....................................................................	6	1	Mm2
....................aaggauguuugacaagCaguuuu.....................................................................	4	1	Mm2
....................aaggauguuugaUaaguaguuuu.....................................................................	1	1	Mm2
....................aaggauguuugacaaguaguuAu.....................................................................	1	1	Mm2
....................aaggauguuugacaaguaguuuG.....................................................................	1	1	Mm2
....................aaggauguuugacaUguaguuuu.....................................................................	1	1	Mm2
....................aaggauguuuUacaaguaguuuu.....................................................................	2	1	Mm2
....................Uaggauguuugacaaguaguuuu.....................................................................	4	1	Mm2
....................aaggauguuugacaagGaguuuu.....................................................................	3	1	Mm2
....................aaggauguuugacaaguGguuuu.....................................................................	11	1	Mm2
....................aaggauguuugaAaaguaguuuu.....................................................................	1	1	Mm2
....................aaggCuguuugacaaguaguuuu.....................................................................	1	1	Mm2
....................Gaggauguuugacaaguaguuuu.....................................................................	3	1	Mm2
....................aCggauguuugacaaguaguuuu.....................................................................	2	1	Mm2
....................aaggauguuugacaaguCguuuu.....................................................................	6	1	Mm2
....................aaggauguuuCacaaguaguuuu.....................................................................	3	1	Mm2
....................Naggauguuugacaaguaguuuu.....................................................................	1	1	Mm2
....................aaggauguuugacaaCuaguuuu.....................................................................	1	1	Mm2
....................aGggauguuugacaaguaguuuu.....................................................................	5	1	Mm2
....................aaggaCguuugacaaguaguuuu.....................................................................	1	1	Mm2
....................aaggauguuugacaaguaUuuuu.....................................................................	1	1	Mm2
....................aaggauguuugacaaguaguuuA.....................................................................	40	1	Mm2
....................aaggauguuugacaaguaguuuuU....................................................................	62	1	Mm2
....................aaggauguuugacaaguaguuuAgu...................................................................	1	1	Mm2
....................aaggauguuugacaaguaguuuuUu...................................................................	3	1	Mm2
.....................aggauguuugacaaguaguu.......................................................................	1	0	Mm2
.....................aggauguuuGcaaguaguuu......................................................................	1	1	Mm2
.....................aggauguuugacaaguaguuu......................................................................	20	0	Mm2
.....................aggauguuugacaaguagGuu......................................................................	1	1	Mm2
.......................gauguuugacaaguaguuu......................................................................	1	0	Mm2
.......................................................acugcuuuuugaacauacuccu...................................	2	0	Mm2
.......................................................acugcuuuuugaacauaAuccuc..................................	1	1	Mm2
.......................................................acugcuuuuugaacauacuccuc..................................	23	0	Mm2
.......................................................acugcuuuuugaacauacuccucU.................................	1	1	Mm2
........................................................cugcuuuuugaacauacuccuc..................................	3	0	Mm2

....................aaggauguuugacaagua..........................................................................	11	0	Mm1
....................aaggauguuugacaaguag.........................................................................	12	0	Mm1
....................aaggauguuugacaaguCgu........................................................................	2	1	Mm1
....................aaggauguuugacaagAagu........................................................................	1	1	Mm1
....................Uaggauguuugacaaguagu........................................................................	2	1	Mm1
....................aaggauguuugacaaguUgu........................................................................	1	1	Mm1
....................aaggauguuugacaagCagu........................................................................	1	1	Mm1
....................aaggauguuugacaagGagu........................................................................	1	1	Mm1
....................aaggauguuugacaaguGgu........................................................................	1	1	Mm1
....................aaggauguuugacaaguagu........................................................................	331	0	Mm1
....................aGggauguuugacaaguagu........................................................................	6	1	Mm1
....................aaggauguuugCcaaguaguu.......................................................................	1	1	Mm1
....................aaggauguuugacaagGaguu.......................................................................	3	1	Mm1
....................aaggauguuugacaaguUguu.......................................................................	2	1	Mm1
....................aaggauguuuCacaaguaguu.......................................................................	1	1	Mm1
....................aaggauguuugacaaguaguG.......................................................................	1	1	Mm1
....................aaggauguuuAacaaguaguu.......................................................................	3	1	Mm1
....................aaggauguuugacaagAaguu.......................................................................	5	1	Mm1
....................aaCgauguuugacaaguaguu.......................................................................	1	1	Mm1
....................aaggauguuugUcaaguaguu.......................................................................	1	1	Mm1
....................Gaggauguuugacaaguaguu.......................................................................	8	1	Mm1
....................Naggauguuugacaaguaguu.......................................................................	1	1	Mm1
```

```
                    Mature                               Star
aaccuugguauaugaaacguaaggauguuugacaaguaguuuugugcgucgacaaacugcuuuuugaacauacuccucguuucuuuaccgagucugcuuauugcuucacgug
....................aaggauguuugacaGguaguu.......................................................................   1      1   Mm1
....................aGggauguuugacaaguaguu.......................................................................   7      1   Mm1
....................aaggauguuugacaaguGguu.......................................................................   5      1   Mm1
....................aaggauguuugacaaguCguu.......................................................................   6      1   Mm1
....................aaggauAuuugacaaguaguu.......................................................................   1      1   Mm1
....................Uaggauguuugacaaguaguu.......................................................................   5      1   Mm1
....................aaggauguuuUacaaguaguu.......................................................................   2      1   Mm1
....................aaggauUuuugacaaguaguu.......................................................................   1      1   Mm1
....................aaggauguuugacUaguaguu.......................................................................   2      1   Mm1
....................aaggauguuugacaaguaguu.......................................................................   1391   0   Mm1
....................aaggauguuugacaagCaguu.......................................................................   3      1   Mm1
....................aaggauguuugacaUguaguu.......................................................................   1      1   Mm1
....................aaggauguuugacaagGaguuu......................................................................   8      1   Mm1
....................aaggauguuugacaaguaguuA......................................................................   32     1   Mm1
....................aaggauguuugacaaguaUuuu......................................................................   1      1   Mm1
....................aaggauguuugacaaguagCuu......................................................................   1      1   Mm1
....................aaggauguCugacaaguaguuu......................................................................   2      1   Mm1
....................aaggauguuugGcaaguaguuu......................................................................   1      1   Mm1
....................aaggaNguuugacaaguaguuu......................................................................   1      1   Mm1
....................Uaggauguuugacaaguaguuu......................................................................   8      1   Mm1
....................aaggauguuugacaaguaguuG......................................................................   2      1   Mm1
....................aaggauguuugacaaguaguCu......................................................................   4      1   Mm1
....................aaggUuguuugacaaguaguuu......................................................................   1      1   Mm1
....................aaggauguuuAacaaguaguuu......................................................................   8      1   Mm1
....................aaggauguuugacaUguaguuu......................................................................   2      1   Mm1
....................aaggauguuugacaaguaguuu......................................................................   6325   0   Mm1
....................aaggauguuCgacaaguaguuu......................................................................   2      1   Mm1
....................aGggauguuugacaaguaguuu......................................................................   5      1   Mm1
....................aaggauguuuCacaaguaguuu......................................................................   10     1   Mm1
....................aagUauguuugacaaguaguuu......................................................................   1      1   Mm1
....................aaggauguuugacUaguaguuu......................................................................   1      1   Mm1
....................aaggauguuugaUaaguaguuu......................................................................   2      1   Mm1
....................aaggauguuuUacaaguaguuu......................................................................   4      1   Mm1
....................aagAauguuugacaaguaguuu......................................................................   2      1   Mm1
....................aaggauguuugacGaguaguuu......................................................................   4      1   Mm1
....................aaggaugCuugacaaguaguuu......................................................................   1      1   Mm1
....................Naggauguuugacaaguaguuu......................................................................   1      1   Mm1
....................aaggauguuugacaaguCguuu......................................................................   19     1   Mm1
....................aaggauguuugacaagCaguuu......................................................................   15     1   Mm1
....................aaggauguuugacaGguaguuu......................................................................   2      1   Mm1
....................aaggauguuugacaaguGguuu......................................................................   24     1   Mm1
....................aaggauguuugaAaaguaguuu......................................................................   1      1   Mm1
....................aaNgauguuugacaaguaguuu......................................................................   1      1   Mm1
....................aaggauguAugacaaguaguuu......................................................................   1      1   Mm1
....................aagCauguuugacaaguaguuu......................................................................   1      1   Mm1
....................aaCgauguuugacaaguaguuu......................................................................   1      1   Mm1
....................aaggauguuugacaaguUguuu......................................................................   17     1   Mm1
....................aaggaugAuugacaaguaguuu......................................................................   1      1   Mm1
....................aaAgauguuugacaaguaguuu......................................................................   3      1   Mm1
....................aaggCuguuugacaaguaguuu......................................................................   1      1   Mm1
....................Gaggauguuugacaaguaguuu......................................................................   7      1   Mm1
....................aaggaAguuugacaaguaguuu......................................................................   1      1   Mm1
....................aUggauguuugacaaguaguuu......................................................................   1      1   Mm1
....................aaggauguuugacaagAaguuu......................................................................   8      1   Mm1
....................aaggauguuugacaGguaguuuu.....................................................................   2      1   Mm1
....................aaggauguuugacaaguaguuuA.....................................................................   6      1   Mm1
....................aaggauguuuCacaaguaguuuu.....................................................................   1      1   Mm1
....................aaggauguuugacaaguaguuCu.....................................................................   1      1   Mm1
....................aaggauguuugacaagCaguuuu.....................................................................   2      1   Mm1
....................aUggauguuugacaaguaguuuu.....................................................................   1      1   Mm1
....................aaggauguuugacaagGaguuuu.....................................................................   1      1   Mm1
....................aaggCuguuugacaaguaguuuu.....................................................................   1      1   Mm1
....................aaggaAguuugacaaguaguuuu.....................................................................   1      1   Mm1
....................Caggauguuugacaaguaguuuu.....................................................................   1      1   Mm1
....................aaggauguuugacaagAaguuuu.....................................................................   2      1   Mm1
....................aaggauguuugacaaguagAuuu.....................................................................   1      1   Mm1
....................aaggauguuugacaaguaguuuu.....................................................................   362    0   Mm1
....................aaggauguuuAacaaguaguuuu.....................................................................   1      1   Mm1
....................aaggauguuugacaaguCguuuu.....................................................................   1      1   Mm1
....................aaggauguuugacaaguaguuuuU....................................................................   16     1   Mm1
```

```
                     Mature                           Star
aaccuugguauaugaaacguaaggauguuugacaaguaguuuugugcgucgacaaacugcuuuuugaacauacuccucguucuuuaccgagucugcuuauugcuucacgug
.....................aggauguuugacaaguaguuu.....................................................................    4     0   Mm1

...................Caaggauguuugacaaguagu.......................................................................    3     1   Fm1
...................uaaggauguuugacaaguaguu......................................................................    3     0   Fm1
...................Caaggauguuugacaaguaguu......................................................................    2     1   Fm1
...................Caaggauguuugacaaguaguuu.....................................................................    3     1   Fm1
...................Caaggauguuugacaaguaguuuu....................................................................    2     1   Fm1
....................aaggauguuugacaaguU.........................................................................    8     1   Fm1
....................aaggauguuugacaaguG.........................................................................    1     1   Fm1
....................aaggauguuugacaagua.........................................................................    117   0   Fm1
....................aaggauguuugacaaguaU........................................................................    4     1   Fm1
....................aaggauguuugacaagCag........................................................................    1     1   Fm1
....................aaggauguuugacaaguag........................................................................    64    0   Fm1
....................aaggauguuugacaaguaA........................................................................    5     1   Fm1
....................aaggauguuuAacaaguagu.......................................................................    1     1   Fm1
....................aaggauguuugacaaguagG.......................................................................    2     1   Fm1
....................aaggauguuugacaagAagu.......................................................................    7     1   Fm1
....................aaggUuguuugacaaguagu.......................................................................    1     1   Fm1
....................aaggauguuuCacaaguagu.......................................................................    2     1   Fm1
....................aaggauguuugacaaguagu.......................................................................    1026  0   Fm1
....................aaggauguuugacaagGagu.......................................................................    2     1   Fm1
....................Uaggauguuugacaaguagu.......................................................................    2     1   Fm1
....................aaggauguuugacaaguagA.......................................................................    13    1   Fm1
....................Gaggauguuugacaaguagu.......................................................................    2     1   Fm1
....................aaggauguuugacaaguaUu.......................................................................    6     1   Fm1
....................aaggauguuugacaaguUgu.......................................................................    3     1   Fm1
....................aaggauguuugaUaaguagu.......................................................................    1     1   Fm1
....................aaggauguuugacaagCagu.......................................................................    6     1   Fm1
....................aCggauguuugacaaguagu.......................................................................    1     1   Fm1
....................aaggauguuugacUaguagu.......................................................................    1     1   Fm1
....................aaggauguuuUacaaguagu.......................................................................    2     1   Fm1
....................aaggauguuugacaaguCgu.......................................................................    3     1   Fm1
....................Naggauguuugacaaguagu.......................................................................    1     1   Fm1
....................aaggauguuugacaaguGgu.......................................................................    3     1   Fm1
....................aaggauguCugacaaguagu.......................................................................    1     1   Fm1
....................aaggauguuugacaagCaguu......................................................................    27    1   Fm1
....................aaggauguuuCacaaguaguu......................................................................    8     1   Fm1
....................aGggauguuugacaaguaguu......................................................................    16    1   Fm1
....................aaggauguuugacGaguaguu......................................................................    1     1   Fm1
....................aaggauguuugacaaguaguG......................................................................    8     1   Fm1
....................aaggauguuugacaaguUguu......................................................................    17    1   Fm1
....................aaggauguuugacaaguGguu......................................................................    17    1   Fm1
....................aaggauguuugacaagAaguu......................................................................    17    1   Fm1
....................Gaggauguuugacaaguaguu......................................................................    8     1   Fm1
....................aaggauguuugacaaguagAu......................................................................    1     1   Fm1
....................aaggauguuugacCaguaguu......................................................................    1     1   Fm1
....................aaggaCguuugacaaguaguu......................................................................    1     1   Fm1
....................aaggauguuuAacaaguaguu......................................................................    11    1   Fm1
....................aaggaugCuugacaaguaguu......................................................................    1     1   Fm1
....................aaggauguuugacaaguaguu......................................................................    5946  0   Fm1
....................aaggauguuuUacaaguaguu......................................................................    2     1   Fm1
....................aaggauguuugacaaguagCu......................................................................    2     1   Fm1
....................aUggauguuugacaaguaguu......................................................................    1     1   Fm1
....................Uaggauguuugacaaguaguu......................................................................    13    1   Fm1
....................Naggauguuugacaaguaguu......................................................................    1     1   Fm1
....................aaggauguuugacUaguaguu......................................................................    2     1   Fm1
....................aaggauguuugUcaaguaguu......................................................................    2     1   Fm1
....................aaggauguuugaUaaguaguu......................................................................    1     1   Fm1
....................aaggauguAugacaaguaguu......................................................................    1     1   Fm1
....................aagCauguuugacaaguaguu......................................................................    1     1   Fm1
....................aaggauguuugacaaguaguC......................................................................    1     1   Fm1
....................aaggauguuugacaaguCguu......................................................................    29    1   Fm1
....................aaggauguuugacaCguaguu......................................................................    1     1   Fm1
....................aaggUuguuugacaaguaguu......................................................................    1     1   Fm1
....................aaggauguuugacaaguaguA......................................................................    20    1   Fm1
....................aaggGuguuugacaaguaguu......................................................................    2     1   Fm1
....................aaggauguuugacaUguaguu......................................................................    1     1   Fm1
....................aaggauguGugacaaguaguu......................................................................    1     1   Fm1
....................aaggauguuugacaagGaguu......................................................................    19    1   Fm1
....................aaggauguuCgacaaguaguu......................................................................    1     1   Fm1
```

```
                    Mature                  Star
aaccuugguauaugaaacguaaggauguuugacaaguaguuuugugcgucgacaaacugcuuuuugaacauacuccucguuucuuuaccgagucugcuuauugcuucacgug
....................aaggauguuugacaUguaguuu......................................................................  9      1  Fm1
....................aaggauguuugacaagGaguuu......................................................................  129    1  Fm1
....................aaggauguuugacaaCuaguuu......................................................................  2      1  Fm1
....................aaggauguuugacaGguaguuu......................................................................  3      1  Fm1
....................aaggUuguuugacaaguaguuu......................................................................  6      1  Fm1
....................aaggGuguuugacaaguaguuu......................................................................  10     1  Fm1
....................aaggauguuuCacaaguaguuu......................................................................  38     1  Fm1
....................aaggauguuugCcaaguaguuu......................................................................  2      1  Fm1
....................aaggauguuugacaaguGguuu......................................................................  130    1  Fm1
....................aaggaugGuugacaaguaguuu......................................................................  3      1  Fm1
....................aaggauguuugacaaguaguuu......................................................................  47586  0  Fm1
....................aaggauguuugacaaguaguAu......................................................................  5      1  Fm1
....................aaggauguuugacaaAuaguuu......................................................................  2      1  Fm1
....................aaggauguuugacaagCaguuu......................................................................  145    1  Fm1
....................aaggaugAuugacaaguaguuu......................................................................  5      1  Fm1
....................aagCauguuugacaaguaguuu......................................................................  2      1  Fm1
....................aaggauguuugaUaaguaguuu......................................................................  16     1  Fm1
....................aaUgauguuugacaaguaguuu......................................................................  10     1  Fm1
....................aaggauNuuugacaaguaguuu......................................................................  2      1  Fm1
....................aaggauguuugacaaguaguCu......................................................................  3      1  Fm1
....................Uaggauguuugacaaguaguuu......................................................................  93     1  Fm1
....................aaggauAuuugacaaguaguuu......................................................................  2      1  Fm1
....................aaggauguuugacaaguagCuu......................................................................  2      1  Fm1
....................aaggauguuCgacaaguaguuu......................................................................  7      1  Fm1
....................aaggauguuugacaaguagAuu......................................................................  4      1  Fm1
....................aaggauguAugacaaguaguuu......................................................................  3      1  Fm1
....................aaCgauguuugacaaguaguuu......................................................................  2      1  Fm1
....................aaggauguuugaAaaguaguuu......................................................................  3      1  Fm1
....................aaggCuguuugacaaguaguuu......................................................................  12     1  Fm1
....................aaggauCuuugacaaguaguuu......................................................................  2      1  Fm1
....................aaggaNguuugacaaguaguuu......................................................................  10     1  Fm1
....................aaggaugCuugacaaguaguuu......................................................................  3      1  Fm1
....................Caggauguuugacaaguaguuu......................................................................  3      1  Fm1
....................aaggauguCugacaaguaguuu......................................................................  4      1  Fm1
....................aaggauguuuUacaaguaguuu......................................................................  34     1  Fm1
....................aaggauguuugacaagAaguuu......................................................................  91     1  Fm1
....................aaggauguuugacaaguCguuu......................................................................  187    1  Fm1
....................aaggauguuugacaaguaAuuu......................................................................  1      1  Fm1
....................aaggauguuugacaaguaUuuu......................................................................  4      1  Fm1
....................aaggauUuuugacaaguaguuu......................................................................  3      1  Fm1
....................aaggauguuGgacaaguaguuu......................................................................  5      1  Fm1
....................aagAauguuugacaaguaguuu......................................................................  6      1  Fm1
....................aaggauguuugUcaaguaguuu......................................................................  11     1  Fm1
....................aUggauguuugacaaguaguuu......................................................................  14     1  Fm1
....................aaggauguuugacaaguaguuG......................................................................  44     1  Fm1
....................aaggaCguuugacaaguaguuu......................................................................  4      1  Fm1
....................aaggauguuugacUaguaguuu......................................................................  6      1  Fm1
....................aaggauguuAgacaaguaguuu......................................................................  1      1  Fm1
....................aaggauguuugacaaguaguuA......................................................................  129    1  Fm1
....................aaggauguuugacaaguagGuu......................................................................  6      1  Fm1
....................aaNgauguuugacaaguaguuu......................................................................  1      1  Fm1
....................aaggauguuugacGaguaguuu......................................................................  12     1  Fm1
....................aaggaGguuugacaaguaguuu......................................................................  2      1  Fm1
....................Gaggauguuugacaaguaguuu......................................................................  46     1  Fm1
....................aaggauguuugacaaguaguGu......................................................................  2      1  Fm1
....................aNggauguuugacaaguaguuu......................................................................  1      1  Fm1
....................aaggauguuugacaaguUguuu......................................................................  140    1  Fm1
....................Naggauguuugacaaguaguuu......................................................................  20     1  Fm1
....................aGggauguuugacaaguaguuu......................................................................  116    1  Fm1
....................aaAgauguuugacaaguaguuu......................................................................  7      1  Fm1
....................aaggauguuugacaCguaguuu......................................................................  2      1  Fm1
....................aaggauguuugacCaguaguuu......................................................................  3      1  Fm1
....................aaggauguuugGcaaguaguuu......................................................................  3      1  Fm1
....................aagUauguuugacaaguaguuu......................................................................  2      1  Fm1
....................aaggauguGugacaaguaguuu......................................................................  2      1  Fm1
....................aaggauguuugacaaguaguuC......................................................................  7      1  Fm1
....................aCggauguuugacaaguaguuu......................................................................  3      1  Fm1
....................aaggaAguuugacaaguaguuu......................................................................  4      1  Fm1
....................aaggauguuuAacaaguaguuu......................................................................  74     1  Fm1
....................aaggauUuuugacaaguaguuuu.....................................................................  1      1  Fm1
```

```
                    Mature                             Star
aaccuugguauaugaaacguaaggauguuugacaaguaguuuugugcgucgacaaacugcuuuuugaacauacuccucguucuuuaccgagucugcuuauugcuucacgug
....................aaggauguuugacaagCaguuuu....................................................................  12    1  Fm1
....................aaggauguuugacUaguaguuuu....................................................................  3     1  Fm1
....................aaAgauguuugacaaguaguuuu....................................................................  1     1  Fm1
....................aaggauguuuUacaaguaguuuu....................................................................  2     1  Fm1
....................aaggauguuugacaaguagGuuu....................................................................  2     1  Fm1
....................aaggGuguuugacaaguaguuuu....................................................................  2     1  Fm1
....................aaggauguuugacaaguCguuuu....................................................................  17    1  Fm1
....................aGggauguuugacaaguaguuuu....................................................................  1     1  Fm1
....................aaggauguuugUcaaguaguuuu....................................................................  2     1  Fm1
....................aaggauguuugacaaguaguuuC....................................................................  3     1  Fm1
....................aaggauguuugacaaguaguuuG....................................................................  4     1  Fm1
....................aaggauguuugacaaguaguuCu....................................................................  1     1  Fm1
....................aaggauguAugacaaguaguuuu....................................................................  2     1  Fm1
....................aaggauguuCgacaaguaguuuu....................................................................  1     1  Fm1
....................aaggauguuugCcaaguaguuuu....................................................................  1     1  Fm1
....................aaggauguuuAacaaguaguuuu....................................................................  4     1  Fm1
....................aaggauguuugacaagGaguuuu....................................................................  3     1  Fm1
....................aaggauguuugacaUguaguuuu....................................................................  2     1  Fm1
....................aaggauguuugacaagAaguuuu....................................................................  9     1  Fm1
....................aaggauguuugacaaguGguuuu....................................................................  14    1  Fm1
....................aaggauguGugacaaguaguuuu....................................................................  1     1  Fm1
....................aaggauguuugacaaguaguuuu....................................................................  3791  0  Fm1
....................Uaggauguuugacaaguaguuuu....................................................................  3     1  Fm1
....................aaggauguuuCacaaguaguuuu....................................................................  2     1  Fm1
....................aaggauguuugacaaguaguuAu....................................................................  6     1  Fm1
....................aaggauguuugacaaguaguuuA....................................................................  100   1  Fm1
....................aagCauguuugacaaguaguuuu....................................................................  1     1  Fm1
....................aaggaNguuugacaaguaguuuu....................................................................  1     1  Fm1
....................Gaggauguuugacaaguaguuuu....................................................................  1     1  Fm1
....................Naggauguuugacaaguaguuuu....................................................................  5     1  Fm1
....................aaggauguuugacaaguUguuuu....................................................................  9     1  Fm1
....................aaggCuguuugacaaguaguuuu....................................................................  1     1  Fm1
....................aaggauguuugacaaCuaguuuu....................................................................  1     1  Fm1
....................aaggauguuugacaaguaguuuuA...................................................................  1     1  Fm1
....................aaggauguuugacaaguaguuuuC...................................................................  3     1  Fm1
....................aaggauguuugacaaguaguuuuU...................................................................  139   1  Fm1
....................aaggauguuugacaaguaguuuuUu..................................................................  14    1  Fm1
.....................aggauguuugUcaaguaguuu.....................................................................  2     1  Fm1
.....................aggaugGuugacaaguaguuu.....................................................................  1     1  Fm1
.....................aggauguuugacaaguaguuu.....................................................................  55    0  Fm1
.....................aggauguuugacaaguaguuuu....................................................................  1     0  Fm1
.......................................................acugcuuuuugaacauacuccuc.................................  21    0  Fm1
.......................................................acugcuuuuugaacauacuccucU................................  3     1  Fm1

...................Caaggauguuugacaaguaguuu.....................................................................  13    1  Fm2
....................aaggauguuugacaagua.........................................................................  31    0  Fm2
....................aaggauguuugacaaguag........................................................................  12    0  Fm2
....................aGggauguuugacaaguag........................................................................  1     1  Fm2
....................aaggauguuugacaaguGgu.......................................................................  1     1  Fm2
....................aaggauguuugacaaguagG.......................................................................  2     1  Fm2
....................Uaggauguuugacaaguagu.......................................................................  2     1  Fm2
....................aaggauguuugacaaguUgu.......................................................................  4     1  Fm2
....................aaggauguuuCacaaguagu.......................................................................  1     1  Fm2
....................aaggauguuugacaaguagA.......................................................................  7     1  Fm2
....................aaggauguuugacaaguCgu.......................................................................  2     1  Fm2
....................aaggauguuugacaagCagu.......................................................................  5     1  Fm2
....................aaggauguuuAacaaguagu.......................................................................  3     1  Fm2
....................aaCgauguuugacaaguagu.......................................................................  1     1  Fm2
....................aaggauguAugacaaguagu.......................................................................  1     1  Fm2
....................aaggauguuugGcaaguagu.......................................................................  1     1  Fm2
....................aaggauguuugacaaguagu.......................................................................  858   0  Fm2
....................aaggauguuCgacaaguagu.......................................................................  1     1  Fm2
....................aaggauguuugacaagAagu.......................................................................  3     1  Fm2
....................aGggauguuugacaaguaguu......................................................................  9     1  Fm2
....................Naggauguuugacaaguaguu......................................................................  1     1  Fm2
....................aaggauguuugacaaguaguu......................................................................  3752  0  Fm2
....................aaggauguuugacaaguaguG......................................................................  2     1  Fm2
....................aaggauguuugaGaaguaguu......................................................................  1     1  Fm2
....................Gaggauguuugacaaguaguu......................................................................  8     1  Fm2
....................aaggauguuugacaaguUguu......................................................................  11    1  Fm2
```

Mature Star

```
aaccuugguauaugaaacguaaggauguuugacaaguaguuuugugcgucgacaaacugcuuuuugaacauacuccucguucuuuaccgagucugcuuauugcuucacgug
....................aaggauguuugacGaguaguu......................................................................  1    1  Fm2
....................aaggauguuugacaaguGguu......................................................................  10   1  Fm2
....................aaggaugGuugacaaguaguu......................................................................  1    1  Fm2
....................aaUgauguuugacaaguaguu......................................................................  1    1  Fm2
....................Uaggauguuugacaaguaguu......................................................................  4    1  Fm2
....................aaggaNguuugacaaguaguu......................................................................  3    1  Fm2
....................aUggauguuugacaaguaguu......................................................................  1    1  Fm2
....................aaggauguuuCacaaguaguu......................................................................  2    1  Fm2
....................aCggauguuugacaaguaguu......................................................................  1    1  Fm2
....................aaggauguuugacaagCaguu......................................................................  11   1  Fm2
....................aaggauguuugacaagAaguu......................................................................  11   1  Fm2
....................aaggauguuuUacaaguaguu......................................................................  2    1  Fm2
....................aaggauguuugacaagGaguu......................................................................  11   1  Fm2
....................aaggauguuugaUaaguaguu......................................................................  1    1  Fm2
....................aaggauguuugacaUguaguu......................................................................  1    1  Fm2
....................aaggCuguuugacaaguaguu......................................................................  3    1  Fm2
....................aaggGuguuugacaaguaguu......................................................................  3    1  Fm2
....................aaggauguuugacaaguaguA......................................................................  14   1  Fm2
....................aaggauguuugacaaguCguu......................................................................  19   1  Fm2
....................aaggauguuuAacaaguaguu......................................................................  4    1  Fm2
....................aagCauguuugacaaguaguu......................................................................  1    1  Fm2
....................aaggauUuuugacaaguaguu......................................................................  1    1  Fm2
....................aaggauguuugUcaaguaguu......................................................................  1    1  Fm2
....................aaggauguuugacaaguNguuu.....................................................................  1    1  Fm2
....................aaggauguuugacaagCaguuu.....................................................................  143  1  Fm2
....................aaggaAguuugacaaguaguuu.....................................................................  5    1  Fm2
....................aaCgauguuugacaaguaguuu.....................................................................  3    1  Fm2
....................aaggauguuCgacaaguaguuu.....................................................................  4    1  Fm2
....................aaggauguuugacaGguaguuu.....................................................................  8    1  Fm2
....................aaggauguAugacaaguaguuu.....................................................................  7    1  Fm2
....................aaggaugCuugacaaguaguuu.....................................................................  6    1  Fm2
....................aaggauguuugaUaaguaguuu.....................................................................  9    1  Fm2
....................aNggauguuugacaaguaguuu.....................................................................  1    1  Fm2
....................aaggauCuuugacaaguaguuu.....................................................................  1    1  Fm2
....................aaggauguuugGcaaguaguuu.....................................................................  2    1  Fm2
....................aaggauguuAgacaaguaguuu.....................................................................  5    1  Fm2
....................aaggauguuugacUaguaguuu.....................................................................  5    1  Fm2
....................aaUgauguuugacaaguaguuu.....................................................................  11   1  Fm2
....................aaggauguuugacaagGaguuu.....................................................................  90   1  Fm2
....................aaggauguuuAacaaguaguuu.....................................................................  66   1  Fm2
....................aaggGuguuugacaaguaguuu.....................................................................  10   1  Fm2
....................aaggauguuugCcaaguaguuu.....................................................................  1    1  Fm2
....................aaggaCguuugacaaguaguuu.....................................................................  5    1  Fm2
....................aaggauguuuUacaaguaguuu.....................................................................  39   1  Fm2
....................aaggauguuugacGaguaguuu.....................................................................  3    1  Fm2
....................aaggauguGugacaaguaguuu.....................................................................  3    1  Fm2
....................aaggauguuugacaaguaguuG.....................................................................  21   1  Fm2
....................aaggauNuuugacaaguaguuu.....................................................................  1    1  Fm2
....................aGggauguuugacaaguaguuu.....................................................................  148  1  Fm2
....................aaggauguuugacaCguaguuu.....................................................................  1    1  Fm2
....................aaggauguuuCacaaguaguuu.....................................................................  24   1  Fm2
....................Caggauguuugacaaguaguuu.....................................................................  3    1  Fm2
....................aaggauguuugacaaguagAuu.....................................................................  4    1  Fm2
....................aaggauguuugacaaguaguuC.....................................................................  1    1  Fm2
....................aaggaugGuugacaaguaguuu.....................................................................  1    1  Fm2
....................aagAauguuugacaaguaguuu.....................................................................  2    1  Fm2
....................aagCauguuugacaaguaguuu.....................................................................  3    1  Fm2
....................Naggauguuugacaaguaguuu.....................................................................  18   1  Fm2
....................aaggauguuugacaaguaguAu.....................................................................  2    1  Fm2
....................aaggauguuugacaaguaguCu.....................................................................  3    1  Fm2
....................aaggUuguuugacaaguaguuu.....................................................................  12   1  Fm2
....................Gaggauguuugacaaguaguuu.....................................................................  102  1  Fm2
....................aaggauguuugacCaguaguuu.....................................................................  4    1  Fm2
....................aaggauguuugacaaguaguGu.....................................................................  2    1  Fm2
....................aaggauguuugacaaUuaguuu.....................................................................  1    1  Fm2
....................aaggauguuugacaaguaUuuu.....................................................................  3    1  Fm2
....................aaggauguuugacaaguaguuA.....................................................................  153  1  Fm2
....................aCggauguuugacaaguaguuu.....................................................................  6    1  Fm2
....................aaggauAuuugacaaguaguuu.....................................................................  5    1  Fm2
....................aaNgauguuugacaaguaguuu.....................................................................  1    1  Fm2
```

```
                    Mature                Star
aaccuugguauaugaaacguaaggauguuugacaaguaguuuugugcgucgacaaacugcuuuuugaacauacuccucguuucuuuaccgagucugcuuauugcuucacgug
....................aaggauUuuugacaaguaguuu......................................................................    6       1    Fm2
....................aaggauguuugacaaguGguuu......................................................................    109     1    Fm2
....................aaggaNguuugacaaguaguuu......................................................................    3       1    Fm2
....................aaggauguuugacaaguagCuu......................................................................    1       1    Fm2
....................aUggauguuugacaaguaguuu......................................................................    16      1    Fm2
....................aaggauguuugacaaAuaguuu......................................................................    2       1    Fm2
....................aaggauguuugacaagAaguuu......................................................................    80      1    Fm2
....................aaAgauguuugacaaguaguuu......................................................................    5       1    Fm2
....................aaggauguCugacaaguaguuu......................................................................    6       1    Fm2
....................Uaggauguuugacaaguaguuu......................................................................    69      1    Fm2
....................aaggauguuugacaaguCguuu......................................................................    144     1    Fm2
....................aaggauguuugUcaaguaguuu......................................................................    21      1    Fm2
....................aaggauguuugacaaguUguuu......................................................................    75      1    Fm2
....................aaggauguuugacaUguaguuu......................................................................    7       1    Fm2
....................aaggauguuGgacaaguaguuu......................................................................    2       1    Fm2
....................aaggauguuugacaaguaguuu......................................................................    38348   0    Fm2
....................aaggauguuugacaaCuaguuu......................................................................    1       1    Fm2
....................aaggCuguuugacaaguaguuu......................................................................    17      1    Fm2
....................aaggauguuugacaaguagGuu......................................................................    1       1    Fm2
....................aaggaugAuugacaaguaguuu......................................................................    3       1    Fm2
....................aagUauguuugacaaguaguuu......................................................................    2       1    Fm2
....................aaggCuguuugacaaguaguuuu.....................................................................    3       1    Fm2
....................aaggauguuugacaaguUguuuu.....................................................................    12      1    Fm2
....................Gaggauguuugacaaguaguuuu.....................................................................    14      1    Fm2
....................Uaggauguuugacaaguaguuuu.....................................................................    6       1    Fm2
....................aaggauguuugacaaguaguuuu.....................................................................    3887    0    Fm2
....................aaggauguuugaUaaguaguuuu.....................................................................    7       1    Fm2
....................aaggauguuugacaaguaUuuuu.....................................................................    1       1    Fm2
....................aaggGuguuugacaaguaguuuu.....................................................................    2       1    Fm2
....................aaggauguuugacaaguGguuuu.....................................................................    8       1    Fm2
....................aaggauguuugacaaguagGuuu.....................................................................    1       1    Fm2
....................aaggauguuuCacaaguaguuuu.....................................................................    7       1    Fm2
....................aaggauguuugacaagGaguuuu.....................................................................    8       1    Fm2
....................aaggauguuugUcaaguaguuuu.....................................................................    2       1    Fm2
....................aaggaAguuugacaaguaguuuu.....................................................................    1       1    Fm2
....................aaggauguuuAacaaguaguuuu.....................................................................    11      1    Fm2
....................aaggauguuugacaaguaguGuu.....................................................................    1       1    Fm2
....................aaggauguuugacGaguaguuuu.....................................................................    1       1    Fm2
....................aGggauguuugacaaguaguuuu.....................................................................    16      1    Fm2
....................aaggauguuugacaaguaguuGu.....................................................................    5       1    Fm2
....................aaggauguuugacaaguaguuuA.....................................................................    210     1    Fm2
....................aaggauAuuugacaaguaguuuu.....................................................................    1       1    Fm2
....................aaggauguuugacCaguaguuuu.....................................................................    1       1    Fm2
....................aaggauguAugacaaguaguuuu.....................................................................    1       1    Fm2
....................aaggauguuGgacaaguaguuuu.....................................................................    1       1    Fm2
....................aaggaugGuugacaaguaguuuu.....................................................................    1       1    Fm2
....................aaggauguuugacaaguaguuCu.....................................................................    1       1    Fm2
....................aaggauguuugacaagAaguuuu.....................................................................    8       1    Fm2
....................aaggauguuugacaaguCguuuu.....................................................................    11      1    Fm2
....................aaUgauguuugacaaguaguuuu.....................................................................    2       1    Fm2
....................aaggauguuugacaaguagAuuu.....................................................................    1       1    Fm2
....................aaggauguuugacaagCaguuuu.....................................................................    11      1    Fm2
....................aaggauguuugacaaguaguuAu.....................................................................    15      1    Fm2
....................aaggauguuAgacaaguaguuuu.....................................................................    2       1    Fm2
....................aaggauguCugacaaguaguuuu.....................................................................    1       1    Fm2
....................aaggauguuuUacaaguaguuuu.....................................................................    2       1    Fm2
....................aaggauguuugacaaguaAuuuu.....................................................................    1       1    Fm2
....................Naggauguuugacaaguaguuuu.....................................................................    4       1    Fm2
....................aaggauguuugacaaguaguuuG.....................................................................    1       1    Fm2
....................aaggaugAuugacaaguaguuuu.....................................................................    1       1    Fm2
....................aaggauguuugacaaguaguuuuU....................................................................    172     1    Fm2
....................aaggauguuugacaaguaguuuuUu...................................................................    19      1    Fm2
.....................aggauguuugacaaguagu........................................................................    1       0    Fm2
.....................aggauguuugacaaguaguu.......................................................................    11      0    Fm2
.....................aAgauguuugacaaguaguu.......................................................................    1       1    Fm2

...................uaaggauguuugacaaguagu........................................................................    1       0    Mm3
...................uaaggauguuugacaaguaguu.......................................................................    1       0    Mm3
...................Caaggauguuugacaaguaguuu......................................................................    1       1    Mm3
...................Caaggauguuugacaaguaguuuu.....................................................................    1       1    Mm3
```

```
                    Mature                            Star
aaccugguauaugaaacguaaggauguuugacaaguaguuuugugcgucgacaaacugcuuuuugaacauacuccucguucuuuaccgagucugcuuauugcuucacgug
....................aaggauguuugacaaguU.........................................................................    1      1    Mm3
....................aaggauguuugacaagua.........................................................................    13     0    Mm3
....................aaggauguuugacaaguag........................................................................    6      0    Mm3
....................aaggauguuugacaaguagA.......................................................................    1      1    Mm3
....................aaggauguuugacaaguUgu.......................................................................    1      1    Mm3
....................aGggauguuugacaaguagu.......................................................................    1      1    Mm3
....................aaggauguuugacaaguCgu.......................................................................    1      1    Mm3
....................aaggauguuugacaaguagu.......................................................................    196    0    Mm3
....................Uaggauguuugacaaguagu.......................................................................    1      1    Mm3
....................aaggauguuugacaagCagu.......................................................................    3      1    Mm3
....................aaggauguuugacaaguaguA......................................................................    3      1    Mm3
....................aCggauguuugacaaguaguu......................................................................    1      1    Mm3
....................aaggGuguuugacaaguaguu......................................................................    1      1    Mm3
....................Uaggauguuugacaaguaguu......................................................................    1      1    Mm3
....................aaggauguuAgacaaguaguu......................................................................    1      1    Mm3
....................aaggauguuuUacaaguaguu......................................................................    1      1    Mm3
....................aaggauguuugacUaguaguu......................................................................    1      1    Mm3
....................aaggauguuugacaaguaguu......................................................................    933    0    Mm3
....................aaggauguuugacaagAaguu......................................................................    1      1    Mm3
....................aaggauguuugacaagCaguu......................................................................    2      1    Mm3
....................aaggauguuugacaaguGguu......................................................................    1      1    Mm3
....................aaggauguuuCacaaguaguu......................................................................    1      1    Mm3
....................Gaggauguuugacaaguaguu......................................................................    2      1    Mm3
....................aGggauguuugacaaguaguu......................................................................    1      1    Mm3
....................aaggauguuugacaaguUguu......................................................................    3      1    Mm3
....................aaggauguuugacaaguCguu......................................................................    3      1    Mm3
....................Caggauguuugacaaguaguuu.....................................................................    1      1    Mm3
....................aaggauguuuUacaaguaguuu.....................................................................    7      1    Mm3
....................aaggauguuugacaagCaguuu.....................................................................    26     1    Mm3
....................aaggauUuuugacaaguaguuu.....................................................................    2      1    Mm3
....................aaggauguuugacCaguaguuu.....................................................................    1      1    Mm3
....................aaggauguuugacaaguaguuC.....................................................................    1      1    Mm3
....................aaAgauguuugacaaguaguuu.....................................................................    2      1    Mm3
....................aaggGuguuugacaaguaguuu.....................................................................    3      1    Mm3
....................aaggauguuugacaaguaguuG.....................................................................    1      1    Mm3
....................Uaggauguuugacaaguaguuu.....................................................................    24     1    Mm3
....................aNggauguuugacaaguaguuu.....................................................................    1      1    Mm3
....................aaggauguuugGcaaguaguuu.....................................................................    1      1    Mm3
....................aaggCuguuugacaaguaguuu.....................................................................    6      1    Mm3
....................aaggauguuugacUaguaguuu.....................................................................    1      1    Mm3
....................aaggauguuugacGaguaguuu.....................................................................    3      1    Mm3
....................aaggauguCugacaaguaguuu.....................................................................    1      1    Mm3
....................aaggauguuugacaaUuaguuu.....................................................................    1      1    Mm3
....................aaggauguuuCacaaguaguuu.....................................................................    3      1    Mm3
....................aaggauguuGgacaaguaguuu.....................................................................    1      1    Mm3
....................aaggauguuugacaaguaguuu.....................................................................    7057   0    Mm3
....................aaggauguuugacaCguaguuu.....................................................................    1      1    Mm3
....................aaggauguuugacaaguaguuA.....................................................................    19     1    Mm3
....................aaggauguuugCcaaguaguuu.....................................................................    1      1    Mm3
....................aaggauguuugacaaguaguAu.....................................................................    1      1    Mm3
....................aCggauguuugacaaguaguuu.....................................................................    1      1    Mm3
....................aaggauguAugacaaguaguuu.....................................................................    1      1    Mm3
....................aaggauguuugacaGguaguuu.....................................................................    1      1    Mm3
....................Naggauguuugacaaguaguuu.....................................................................    3      1    Mm3
....................aaggaugCuugacaaguaguuu.....................................................................    1      1    Mm3
....................aaggauguuugacaagAaguuu.....................................................................    10     1    Mm3
....................aaggauguuugacaagGaguuu.....................................................................    21     1    Mm3
....................aaggauguuuAacaaguaguuu.....................................................................    9      1    Mm3
....................aaggauguuugacaaguUguuu.....................................................................    23     1    Mm3
....................Gaggauguuugacaaguaguuu.....................................................................    11     1    Mm3
....................aaggauguuugacaaguGguuu.....................................................................    14     1    Mm3
....................aGggauguuugacaaguaguuu.....................................................................    21     1    Mm3
....................aaggauguuugacaaguagCuu.....................................................................    1      1    Mm3
....................aaggauguuugUcaaguaguuu.....................................................................    1      1    Mm3
....................aaCgauguuugacaaguaguuu.....................................................................    2      1    Mm3
....................aUggauguuugacaaguaguuu.....................................................................    6      1    Mm3
....................aaggauguuugacaaguCguuu.....................................................................    29     1    Mm3
....................aaggauguuugaUaaguaguuu.....................................................................    1      1    Mm3
....................aaggUuguuugacaaguaguuu.....................................................................    2      1    Mm3
....................aaggaCguuugacaaguaguuu.....................................................................    3      1    Mm3
```

```
                    Mature                              Star
aaccuugguauaugaaacguaaggauguuugacaaguaguuuugugcgucgacaaacugcuuuuugaacauacuccucguucuuuaccgagucugcuuauugcuucacgug
....................aaAgauguuugacaaguaguuuu....................................................................	1	1	Mm3
....................aaggauguuugacaaguaguuuu....................................................................	481	0	Mm3
....................aaggauguuugUcaaguaguuuu....................................................................	1	1	Mm3
....................Uaggauguuugacaaguaguuuu....................................................................	1	1	Mm3
....................aaggauguuugacaagAaguuuu....................................................................	3	1	Mm3
....................aaggauguuugacaaguUguuuu....................................................................	1	1	Mm3
....................Gaggauguuugacaaguaguuuu....................................................................	1	1	Mm3
....................aaggauguuugacaaguaguuAu....................................................................	2	1	Mm3
....................aaggauguuugacaagCaguuuu....................................................................	2	1	Mm3
....................aaggUuguuugacaaguaguuuu....................................................................	2	1	Mm3
....................aaggauguuugacaaguaguuuA....................................................................	23	1	Mm3
....................aGggauguuugacaaguaguuuu....................................................................	2	1	Mm3
....................aaggauguuugacaagGaguuuu....................................................................	1	1	Mm3
....................aaggauguuugacaaUuaguuuu....................................................................	1	1	Mm3
....................aaggauguuugacaaguaguuuuU...................................................................	20	1	Mm3
....................aaggauguuugacaaguaguuuuUu..................................................................	8	1	Mm3
.....................aggauguuugacaaguaguu......................................................................	1	0	Mm3
.....................aggauguuugacaaguaguuu.....................................................................	10	0	Mm3
.....................aUgauguuugacaaguaguuu.....................................................................	1	1	Mm3
.....................aggauguuugacaaguaguuuu....................................................................	1	0	Mm3
.......................Aauguuugacaaguaguuu.....................................................................	1	1	Mm3
.......................................................acugcuuuuugaacauacuccu..................................	4	0	Mm3
.......................................................acugcuuuuugaacauacuccuc.................................	5	0	Mm3

..................guaaggauguuugacaaguaguu......................................................................	1	0	Fm3
...................uaaggauguuugacaagua.........................................................................	1	0	Fm3
...................Caaggauguuugacaagua.........................................................................	4	1	Fm3
...................uaaggauguuugacaaguagu.......................................................................	3	0	Fm3
...................uaaggauguuugacaaguaguu......................................................................	7	0	Fm3
...................Caaggauguuugacaaguaguu......................................................................	6	1	Fm3
...................Caaggauguuugacaaguaguuu.....................................................................	15	1	Fm3
...................Aaaggauguuugacaaguaguuu.....................................................................	1	1	Fm3
....................aaggauguuugacaagua.........................................................................	284	0	Fm3
....................aaggCuguuugacaagua.........................................................................	1	1	Fm3
....................aaggauguuugacaagAa.........................................................................	1	1	Fm3
....................aaggauguuugacaagGa.........................................................................	2	1	Fm3
....................aaggauguuugacaaguU.........................................................................	5	1	Fm3
....................Uaggauguuugacaagua.........................................................................	1	1	Fm3
....................aaggauguuugacaaguG.........................................................................	2	1	Fm3
....................aaggauguuGgacaagua.........................................................................	1	1	Fm3
....................aaggauguuugacaaguag........................................................................	175	0	Fm3
....................aaggauguuugacaaguaA........................................................................	39	1	Fm3
....................aaggauguuugacaaguGg........................................................................	1	1	Fm3
....................aaggauguuugaUaaguag........................................................................	1	1	Fm3
....................aaggauguuuAacaaguag........................................................................	2	1	Fm3
....................aaggauguuugacaagCag........................................................................	2	1	Fm3
....................aaAgauguuugacaaguag........................................................................	1	1	Fm3
....................aaggauguuugacaaguCg........................................................................	1	1	Fm3
....................aaggauguuugacaaguaU........................................................................	15	1	Fm3
....................aaggauguuuUacaaguagu.......................................................................	2	1	Fm3
....................aaggauguuugacaaguagA.......................................................................	15	1	Fm3
....................aaggauUuuugacaaguagu.......................................................................	1	1	Fm3
....................aaggauguuugacaaguUgu.......................................................................	13	1	Fm3
....................aaggauguuugacaagAagu.......................................................................	3	1	Fm3
....................aaggauguuugacGaguagu.......................................................................	1	1	Fm3
....................aaggauguuuCacaaguagu.......................................................................	1	1	Fm3
....................Gaggauguuugacaaguagu.......................................................................	5	1	Fm3
....................Naggauguuugacaaguagu.......................................................................	2	1	Fm3
....................aaggauguuugacaagCagu.......................................................................	15	1	Fm3
....................Uaggauguuugacaaguagu.......................................................................	7	1	Fm3
....................aaggauguuCgacaaguagu.......................................................................	1	1	Fm3
....................aaggauguuugacaaguaUu.......................................................................	1	1	Fm3
....................aagAauguuugacaaguagu.......................................................................	1	1	Fm3
....................aaggauguuugacaagGagu.......................................................................	17	1	Fm3
....................aaggaAguuugacaaguagu.......................................................................	2	1	Fm3
....................aaggGuguuugacaaguagu.......................................................................	2	1	Fm3
....................aUggauguuugacaaguagu.......................................................................	1	1	Fm3
....................aaggauguuugaGaaguagu.......................................................................	2	1	Fm3
....................aaggCuguuugacaaguagu.......................................................................	1	1	Fm3
....................aaUgauguuugacaaguagu.......................................................................	6	1	Fm3
```

```
                    Mature               Star
aaccuugguauaugaaacguaaggauguuugacaaguaguuuugugcgucgacaaacugcuuuuugaacauacuccucguucuuuaccgagucugcuuauugcuucacgug
....................aaggauguuugacaaguCgu.......................................................................    15     1    Fm3
....................aaggauguuuAacaaguagu.......................................................................    7      1    Fm3
....................aaggaugCuugacaaguagu.......................................................................    3      1    Fm3
....................aGggauguuugacaaguagu.......................................................................    6      1    Fm3
....................aCggauguuugacaaguagu.......................................................................    1      1    Fm3
....................aaggauguuugUcaaguagu.......................................................................    4      1    Fm3
....................aaggauguuugGcaaguagu.......................................................................    1      1    Fm3
....................aagCauguuugacaaguagu.......................................................................    1      1    Fm3
....................aaggauguuugacaaguagu.......................................................................    3999   0    Fm3
....................aaggaugAuugacaaguagu.......................................................................    1      1    Fm3
....................aaggauCuuugacaaguagu.......................................................................    1      1    Fm3
....................aaggauguuugacaaguaAu.......................................................................    1      1    Fm3
....................aaggauguuugacaaguGgu.......................................................................    13     1    Fm3
....................aaggauguuugacaaguaguC......................................................................    2      1    Fm3
....................aaggauguuuCacaaguaguu......................................................................    9      1    Fm3
....................aaggauguuugacaaguagAu......................................................................    1      1    Fm3
....................aaggauguuugacaaguCguu......................................................................    68     1    Fm3
....................aaggauNuuugacaaguaguu......................................................................    1      1    Fm3
....................aaggauguuugacaagAaguu......................................................................    39     1    Fm3
....................aaggauguuugacUaguaguu......................................................................    4      1    Fm3
....................Gaggauguuugacaaguaguu......................................................................    7      1    Fm3
....................aaggauguuugacaaAuaguu......................................................................    1      1    Fm3
....................aagUauguuugacaaguaguu......................................................................    3      1    Fm3
....................aaggauAuuugacaaguaguu......................................................................    1      1    Fm3
....................aaggauguuugaGaaguaguu......................................................................    1      1    Fm3
....................Caggauguuugacaaguaguu......................................................................    1      1    Fm3
....................aaggauguuugacaCguaguu......................................................................    1      1    Fm3
....................aaggauguuuAacaaguaguu......................................................................    23     1    Fm3
....................aagCauguuugacaaguaguu......................................................................    1      1    Fm3
....................aaggaugAuugacaaguaguu......................................................................    1      1    Fm3
....................aaggaNguuugacaaguaguu......................................................................    2      1    Fm3
....................aaggauguuugaAaaguaguu......................................................................    2      1    Fm3
....................aaUgauguuugacaaguaguu......................................................................    6      1    Fm3
....................aaggauguuCgacaaguaguu......................................................................    2      1    Fm3
....................aaAgauguuugacaaguaguu......................................................................    2      1    Fm3
....................aaggauCuuugacaaguaguu......................................................................    2      1    Fm3
....................aaggauguuugaUaaguaguu......................................................................    9      1    Fm3
....................aaggauguuGgacaaguaguu......................................................................    1      1    Fm3
....................Uaggauguuugacaaguaguu......................................................................    21     1    Fm3
....................aaggUuguuugacaaguaguu......................................................................    2      1    Fm3
....................aaggauguuugacaaguaUuu......................................................................    1      1    Fm3
....................aaggauguuugacaaguaAuu......................................................................    3      1    Fm3
....................aaggaugCuugacaaguaguu......................................................................    2      1    Fm3
....................aaggauguuugacaGguaguu......................................................................    2      1    Fm3
....................aaggauguuugacaaguaguA......................................................................    65     1    Fm3
....................aaggauguuugacaaguUguu......................................................................    22     1    Fm3
....................aaggauguAugacaaguaguu......................................................................    2      1    Fm3
....................aaggauguuugacaaguGguu......................................................................    39     1    Fm3
....................aGggauguuugacaaguaguu......................................................................    11     1    Fm3
....................aUggauguuugacaaguaguu......................................................................    2      1    Fm3
....................aaggauguuugacaUguaguu......................................................................    3      1    Fm3
....................aaggauguuugacaaguagCu......................................................................    2      1    Fm3
....................aaggGuguuugacaaguaguu......................................................................    1      1    Fm3
....................aaggauguuAgacaaguaguu......................................................................    3      1    Fm3
....................aaggauguuugacaagGaguu......................................................................    46     1    Fm3
....................aaggauguuuUacaaguaguu......................................................................    12     1    Fm3
....................aaggCuguuugacaaguaguu......................................................................    5      1    Fm3
....................aaggauguGugacaaguaguu......................................................................    1      1    Fm3
....................aCggauguuugacaaguaguu......................................................................    1      1    Fm3
....................aaggauguuugacaaguaguu......................................................................    14292  0    Fm3
....................aaggauguuugacaagCaguu......................................................................    55     1    Fm3
....................aaggauguuugacCaguaguu......................................................................    1      1    Fm3
....................aaggauguuugUcaaguaguu......................................................................    7      1    Fm3
....................aaCgauguuugacaaguaguu......................................................................    1      1    Fm3
....................aagAauguuugacaaguaguu......................................................................    2      1    Fm3
....................Naggauguuugacaaguaguu......................................................................    3      1    Fm3
....................aaggauguuugacGaguaguu......................................................................    2      1    Fm3
....................aaggauguuugacaagGaguuu.....................................................................    119    1    Fm3
....................aaggauUuuugacaaguaguuu.....................................................................    2      1    Fm3
....................aaggauguuugaAaaguaguuu.....................................................................    1      1    Fm3
```

```
                    Mature                        Star
aaccuugguauaugaaacguaaggauguuugacaaguaguuuugugcgucgacaaacugcuuuuugaacauacuccucguucuuuaccgagucugcuuauugcuucacgug
....................aaggauguuugacGaguaguuu.....................................................................    15      1    Fm3
....................aaggauguuAgacaaguaguuu.....................................................................    2       1    Fm3
....................aaggauguuugacaaAuaguuu.....................................................................    4       1    Fm3
....................aagCauguuugacaaguaguuu.....................................................................    1       1    Fm3
....................aaggauguuugacaagCaguuu.....................................................................    160     1    Fm3
....................aaggauguuugacUaguaguuu.....................................................................    10      1    Fm3
....................aaggauguuugacaaCuaguuu.....................................................................    1       1    Fm3
....................aaAgauguuugacaaguaguuu.....................................................................    12      1    Fm3
....................aaggauguCugacaaguaguuu.....................................................................    3       1    Fm3
....................aaggauguuugacaaguaguCu.....................................................................    1       1    Fm3
....................aaNgauguuugacaaguaguuu.....................................................................    1       1    Fm3
....................aaggauguuugacaaguUguuu.....................................................................    105     1    Fm3
....................aaggaAguuugacaaguaguuu.....................................................................    1       1    Fm3
....................aaggauguuugaGaaguaguuu.....................................................................    4       1    Fm3
....................aaggauguGugacaaguaguuu.....................................................................    3       1    Fm3
....................aaggauguuugacaaguaguAu.....................................................................    5       1    Fm3
....................aaggauguuugacaaguGguuu.....................................................................    132     1    Fm3
....................aaggauguuugacaGguaguuu.....................................................................    4       1    Fm3
....................aaggauguuugacaaguCguuu.....................................................................    173     1    Fm3
....................Gaggauguuugacaaguaguuu.....................................................................    22      1    Fm3
....................aaggauguuugacaaUuaguuu.....................................................................    11      1    Fm3
....................aaggauguuCgacaaguaguuu.....................................................................    7       1    Fm3
....................aaggauguuugacaUguaguuu.....................................................................    6       1    Fm3
....................aCggauguuugacaaguaguuu.....................................................................    7       1    Fm3
....................aaggaugAuugacaaguaguuu.....................................................................    2       1    Fm3
....................aaggauguuugacaaguaAuuu.....................................................................    1       1    Fm3
....................aUggauguuugacaaguaguuu.....................................................................    11      1    Fm3
....................aaggauguuugGcaaguaguuu.....................................................................    4       1    Fm3
....................aaggauNuuugacaaguaguuu.....................................................................    4       1    Fm3
....................aaggauguuugacaaguagCuu.....................................................................    5       1    Fm3
....................aaggauguuugacaagAaguuu.....................................................................    103     1    Fm3
....................aaggauguuugacaaguaUuuu.....................................................................    1       1    Fm3
....................aaggauguuugUcaaguaguuu.....................................................................    9       1    Fm3
....................aaggauguuugacaaguaguuu.....................................................................    47728   0    Fm3
....................aagUauguuugacaaguaguuu.....................................................................    3       1    Fm3
....................Uaggauguuugacaaguaguuu.....................................................................    99      1    Fm3
....................aaggauguuugacaaguaguuG.....................................................................    1       1    Fm3
....................aaggauguuGgacaaguaguuu.....................................................................    9       1    Fm3
....................aaggGuguuugacaaguaguuu.....................................................................    15      1    Fm3
....................aaggauguuugaUaaguaguuu.....................................................................    11      1    Fm3
....................aaggauguuugacaaguaguGu.....................................................................    4       1    Fm3
....................aaggauguuugacaaguagGuu.....................................................................    3       1    Fm3
....................aaUgauguuugacaaguaguuu.....................................................................    15      1    Fm3
....................aaggaCguuugacaaguaguuu.....................................................................    3       1    Fm3
....................aaggauguuugacaaguaguuC.....................................................................    14      1    Fm3
....................aaggauguuuUacaaguaguuu.....................................................................    32      1    Fm3
....................aaggUuguuugacaaguaguuu.....................................................................    4       1    Fm3
....................aaCgauguuugacaaguaguuu.....................................................................    1       1    Fm3
....................aaggauguuugacaaguagAuu.....................................................................    1       1    Fm3
....................aaggauguuugacCaguaguuu.....................................................................    1       1    Fm3
....................aaggauAuuugacaaguaguuu.....................................................................    4       1    Fm3
....................aaggaNguuugacaaguaguuu.....................................................................    3       1    Fm3
....................aaggauguuuAacaaguaguuu.....................................................................    93      1    Fm3
....................aaggauguAugacaaguaguuu.....................................................................    5       1    Fm3
....................aaggauguuugacaaguaguuA.....................................................................    398     1    Fm3
....................aagAauguuugacaaguaguuu.....................................................................    3       1    Fm3
....................aaggauguuuCacaaguaguuu.....................................................................    55      1    Fm3
....................Naggauguuugacaaguaguuu.....................................................................    27      1    Fm3
....................aaggaugGuugacaaguaguuu.....................................................................    3       1    Fm3
....................aaggaugCuugacaaguaguuu.....................................................................    8       1    Fm3
....................aGggauguuugacaaguaguuu.....................................................................    82      1    Fm3
....................aaggCuguuugacaaguaguuu.....................................................................    11      1    Fm3
....................Caggauguuugacaaguaguuu.....................................................................    7       1    Fm3
....................Uaggauguuugacaaguaguuuu....................................................................    3       1    Fm3
....................aaggauguuugacaaguaguuuu....................................................................    459     0    Fm3
....................aaggauguuuCacaaguaguuuu....................................................................    1       1    Fm3
....................aaggauguuugacaaguCguuuu....................................................................    1       1    Fm3
....................aaggauguuugacaaguUguuuu....................................................................    1       1    Fm3
....................aaggauguuugacaaguagGuuu....................................................................    1       1    Fm3
....................aaggauguuugacaaguaguuuG....................................................................    1       1    Fm3
```

```
                    Mature                            Star
aaccuugguauaugaaacguaaggauguuugacaaguaguuuugugcgucgacaaacugcuuuuugaacauacuccucguuucuuuaccgagucugcuuauugcuucacgug
....................aaggauguuugacaaguGguuuu.....................................................................    2     1     Fm3
....................aaggauguuugacaaguaguuAu.....................................................................    17    1     Fm3
....................aaggauguuuAacaaguaguuuu.....................................................................    2     1     Fm3
....................aaggauguuugacaagAaguuuu.....................................................................    1     1     Fm3
....................aaggCuguuugacaaguaguuuu.....................................................................    1     1     Fm3
....................aaggauguuugacaaguaguuuA.....................................................................    66    1     Fm3
....................aaggauguuugacaagCaguuuu.....................................................................    2     1     Fm3
....................aaggauguuugacaaguaguuCu.....................................................................    1     1     Fm3
....................aaggauguuugacaaguaguuGu.....................................................................    4     1     Fm3
....................aaggauguuugacaaguaguuuuA....................................................................    1     1     Fm3
....................aaggauguuugacaaguaguuuuU....................................................................    2     1     Fm3
.....................aggauguuugacaaguaguu.......................................................................    2     0     Fm3
.....................aggauAuuugacaaguaguuu......................................................................    1     1     Fm3
.....................aggauguuugacaaguCguuu......................................................................    1     1     Fm3
.....................aggauguuuUacaaguaguuu......................................................................    1     1     Fm3
.....................aggauguAugacaaguaguuu......................................................................    1     1     Fm3
.....................aggauguuugacaaguaguuu......................................................................    68    0     Fm3
.....................aggauguuugacaaguagGuu......................................................................    1     1     Fm3
.......................gauguuugacaaguaguuu......................................................................    8     0     Fm3
.......................................................acugcuuuuugaacauacuccuc..................................    2     0     Fm3
```
